# Bicuspid Pulmonic Valve and Pulmonary Artery Aneurysm

**DOI:** 10.14740/cr321w

**Published:** 2014-05-15

**Authors:** Carlos A. Jamis-Dow, George H. Barbier, Mary P. Watkins, Gregory M. Lanza, Shelton D. Caruthers, Samuel A. Wickline

**Affiliations:** a Department of Radiology, Penn State Milton S. Hershey Medical Center, Hershey, Pennsylvania, USA; b Cardiology Section, Bay Pines VA Healthcare System, Bay Pines, Florida, USA; c Consortium for Translational Research in Advanced Imaging and Nanomedicine (C-TRAIN), Washington University of Saint Louis, St Louis, Missouri, USA

**Keywords:** Bicuspid pulmonic valve, Pulmonary artery aneurysm, Neural crest cells

## Abstract

Bicuspid pulmonary valves and pulmonary artery aneurysms are two rare entities, reported in association, and usually attributed to hemodynamic alterations caused by the bicuspid pulmonary valve. We present magnetic resonance images of a patient with a bicuspid pulmonary valve and pulmonary artery aneurysm, and propose an alternative mechanism for this association, based on recent embryologic studies that link anomalies of the semilunar valves and great vessels with derangement of the cardiac neural crest cell development.

## Introduction

Bicuspid pulmonary valves are rare. A study of 3,861 donor hearts, dissected at the European Homograft Bank revealed only four cases (0.1%) of bicuspid pulmonary valves [1]. Pulmonary artery aneurysms are even rarer, with only eight cases documented in 109,571 autopsies [2]. Bicuspid pulmonary valves have been reported in association with pulmonary artery aneurysms [2]. In some cases, the presence of the aneurysm is attributed to the stenotic pulmonary valve. However, another reason for this association may be found in the embryologic development of the great vessels and their semilunar valves. Migrating neural crest cells are necessary for the normal development of the semilunar valves, the septation of the outflow tract (into the aorta and pulmonary artery) and the remodeling of the aortic arch [3]. Using mice with primary and secondary cardiac neural crest deficiencies, Jain et al [3] showed that normal neural crest is necessary for adequate mesenchymal apoptosis in late gestation semilunar valve leaflets, valve remodeling and proper valve architecture. Additionally, they showed that normal neural crest is necessary for the normal development of the smooth muscle layer of the wall of the ascending aorta and aortic arch. Their experimental data provide an explanation for the association between aortic and pulmonary valves defects and vascular abnormalities of the ascending aorta and aortic arch [3]. Since the aorta and pulmonary artery share a common origin, abnormalities of the pulmonary artery may be additional associated findings in patients with semilunar valve defects.

## Case Report

A 59-year-old female with history of an abnormal pulmonary valve volunteered for a routine cardiovascular magnetic resonance (CMR) exam. She was completely asymptomatic and took no medications. She was employed and stated that she had no limitations regarding her exercise capacity. There was no family history of congenital heart disease. CMR was performed in a 1.5 T Philips Achieva whole body MR scanner using a five-element phased-array receive coil (Philips Healthcare, Andover, MA). Breath-hold, balanced steady-state free precession (b-SSFP) cine images were obtained in the vertical long axis, horizontal long axis, left ventricular outflow tract and left ventricular short axis planes. Breath-hold flow quantitation images (2.3 × 2.3 × 10 mm resolution, 150 cm/s through-plane encoding velocity) in planes parallel to the pulmonary valve plane were obtained at the valve level, 1.5 cm above the valve and 1.5 cm below the valve. Images were analyzed using a ViewForum workstation (Philips Healthcare, Andover, MA). Left ventricular ejection fraction was calculated from the short axis images, using Simpson’s rule, excluding the trabeculae and papillary muscles. From the velocity-encoded images, the pulmonary valve area was calculated by planimetry and by applying the continuity equation as previously described (Area_1_ × VTI_1_ = Area_2_ × Peak VTI_2_) [4]. Peak pressure gradient across the pulmonary valve was calculated by using the modified Bernoulli equation (ΔP_peak_ = 4V_peak_^2^).

**Figure 1:**
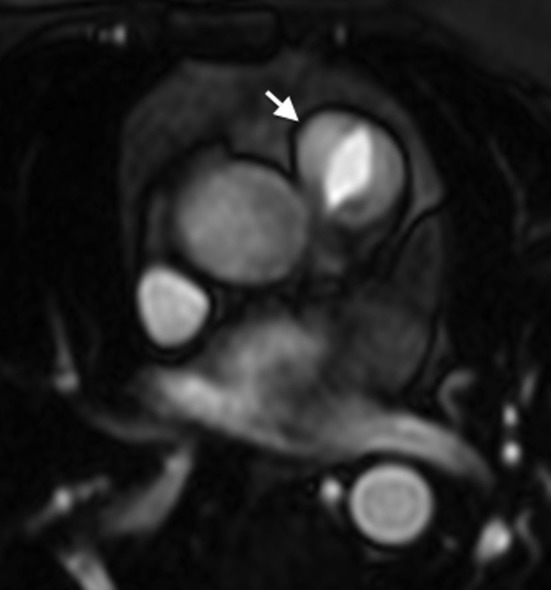
Bicuspid pulmonary valve. b-SSFP image through the proximal pulmonary artery, parallel to the pulmonary valve, showing mildly stenotic bicuspid pulmonary valve (arrow).

**Figure 2:**
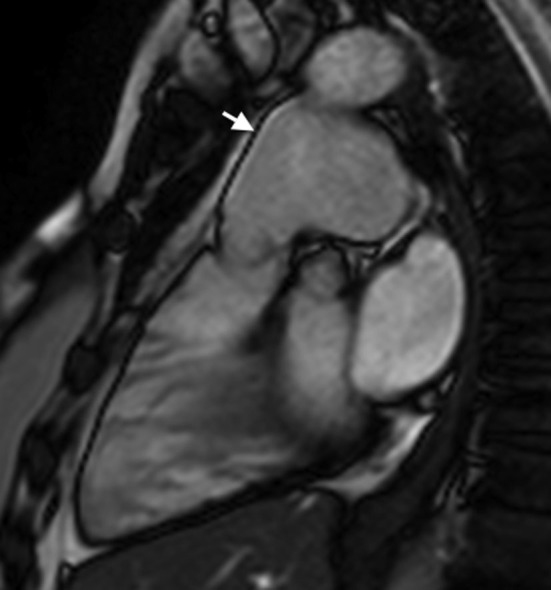
Pulmonary artery aneurysm. Parasagittal b-SSFP image through right ventricular outflow tract, pulmonary valve and main pulmonary artery, showing aneurysmal dilatation of the main pulmonary artery (arrow).

CMR revealed normal right ventricular and left ventricular function, normal aortic valve, bicuspid pulmonary valve and aneurysmal dilatation of the pulmonary artery trunk with a diameter of 3.9 cm (Fig. 1, 2). The pulmonary valve area measured 1.7 cm^2^ by planimetry, and 2.1 cm^2^ using the continuity equation. The calculated peak pressure gradient across the pulmonary valve was 13.5 mm Hg. For video supplementary files, see www.cardiologyres.org.

## Discussion

Given our patient’s mild degree of pulmonary valve stenosis, it is possible that her pulmonary artery aneurysm is not secondary to hemodynamic alterations attributable to her bicuspid pulmonary valve, but that her bicuspid pulmonary valve and pulmonary artery aneurysm may be both secondary to alterations in her normal cardiac neural crest cell development. 
